# Increase in predation risk and trophic level induced by nocturnal visits of piscivorous fishes in a temperate seagrass bed

**DOI:** 10.1038/s41598-017-04217-3

**Published:** 2017-06-20

**Authors:** Jun Shoji, Hiromichi Mitamura, Kotaro Ichikawa, Hikari Kinoshita, Nobuaki Arai

**Affiliations:** 1 Graduate School of Biosphere Science, Hiroshima University, Hiroshima, 739-8528 Japan; 2 Graduate School of Informatics, Kyoto University, Kyoto, 606-8501 Japan; 3 Field Science Education and Research Center, Kyoto University, Kyoto, 606-8502 Japan

## Abstract

The majority of surveys on food webs of aquatic ecosystems have been conducted during the day owning to difficulties in sampling animals at night. In this study, to examine diurnal changes in predator-prey interactions in a temperate seagrass *Zostera marina* bed, a quantitative day/night survey of fish, the dominant animal community, coupled with acoustic telemetry of their predators, was conducted. The number of species, abundance, and biomass of piscivorous predators and mean trophic level during the night were significantly higher than those in the day in all seasons. Analysis of the stomach contents of 182 piscivorous predators showed that no fish predation occurred during the day whereas predation occurred during the night in winter, spring, and summer. Acoustic telemetry demonstrated nocturnal visits by dominant piscivorous fish species (rockfishes and conger eel) to the seagrass bed. We conclude that the nocturnal visits by piscivorous fishes increased the predation risk and trophic level in the fish nursery. The ecological functions of seagrass beds should be reevaluated accounting for day/night changes in food webs; these areas serve as nurseries for juvenile and small-sized fishes during the day and as foraging grounds for predators during the night.

## Introduction

Over an annual cycle, nighttime constitutes half of the diel cycle, on average. Animal community structure and ecosystem function vary within the diel cycle, reflecting the day/night changes in the patterns of distribution and behavior of each species^1, 2^. However, most surveys to evaluate animal communities and species interactions have been conducted during the day owning to difficulties in animal sampling at night. Information on the distribution and behavior of migratory animals during the night is limited, especially in aquatic ecosystems compared with terrestrial ecosystems^3–6^.

**Figure 1:**
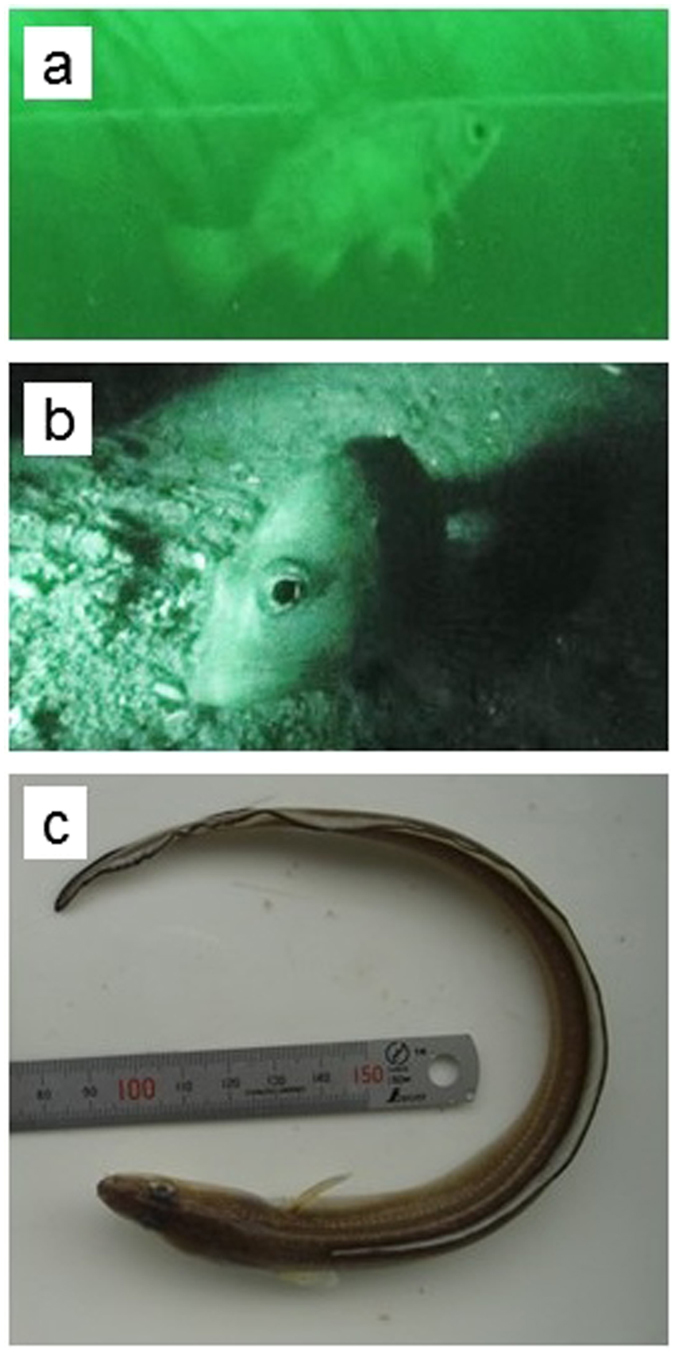
Nocturnal visit of piscivorous fish predators monitored by an underwater video camera with an infra-red light (**a**) black rockfish *Sebastes* sp., (**b**) marbled rockfish *Sebastiscus marmoratus*) and conger eel *Conger myriaster* (**c**) collected from the seagrass bed during the field survey.

Fish are a major component of animal communities, and support trophic flow in aquatic ecosystems. The movement of fishes from one ecosystem to another (e.g., nocturnal visits by piscivorous vertebrate predators; Fig. 1) can cause dramatic changes in species interactions and trophic flow over a short time scale. However, it has been difficult to clarify the trophic flow within a single aquatic ecosystem because many predatory species utilize (i.e., visit and feed in) multiple ecosystems over different time scales, such as a day, season, year, or developmental stage of life^7^. It is essential to clarify how predators utilize multiple habitats during foraging migrations, and the extent of allochthonous resource flow, to comprehensively evaluate the structure and function of marine ecosystems^8^.

Marine coastal areas provide a variety of ecosystem services, and their economic value has been estimated among the highest of the world’s ecosystems^9^. The complexity and connectivity provided by multiple ecosystems, such as seagrass beds, mangroves, and coral reefs, increase biodiversity and productivity^10, 11^. Seagrass beds are important ecosystems in marine coastal areas because of their high productivity and biodiversity^12, 13^. Elevated abundance and biomass of epi-faunal and in-benthic organisms in seagrass beds serve as important food resources for fish predators. Furthermore, the habitat complexity of seagrass beds affects vulnerability to predation, which is the major source of mortality of juvenile and small-sized fishes^14^. In general, seagrass beds are referred to as a fish nursery, because the highly complex vegetation prevents piscivorous fishes from feeding, and thus serves as a predation refuge for juvenile and small-sized fishes^7, 13^. This nursery role is an important component of the ecological functions of the seagrass bed^14^.

However, to date, evaluation of the ecological functions of the seagrass bed, especially as a predation refuge, have used information of the fish community structure and predator-prey interactions obtained during the day^12, 13^. Information obtained during both day and night is indispensable to adequately evaluate the ecological function of a habitat. The results of recent field surveys and experiments have indicated that the common understanding of seagrass beds functioning as predation refuges for small fishes may be only partially correct. The fish community structure and predation rate of juvenile fishes have been shown to differ between daytime and nighttime in seagrass beds of the western Pacific^15–17^. These observations imply that although seagrass beds act as predation refuges for juveniles and small-sized fishes during the day, they may be focal foraging areas for predators during the night. In addition, from the viewpoint of predatory fishes, feeding on juvenile or small-sized fishes enables them to obtain energy, indicating another ecological function of seagrass beds, which contributes to the production of larger migratory fishes through allochthonous resource flow. Therefore, there seems to be a paradox in the ecological functions of the seagrass beds, whereby they serve as a predation refuge for juvenile and small-sized fishes but also as a focal feeding ground for nocturnal piscivorous predators.

To provide a more accurate and comprehensive evaluation of the ecological function of seagrass beds, improvement of survey methods, as well as extension of the survey period (daytime only to whole day) are essential. Data collected by nets and trawls may over/under estimate abundance, especially for large-sized piscivorous fishes, due to differences in catch efficiency at different times of the day. For example, significant changes in the abundance and/or biomass of piscivorous fish predators during the nighttime are commonly observed in seagrass beds^15–17^ and may indicate higher catch efficiency of sampling gears in the dark. Therefore, evidence of nocturnal migration of these predatory fishes would clarify the functions of the seagrass bed as a feeding ground for piscivorous fishes.

Acoustic telemetry using ultrasonic transmitters has been used to examine the behavior and migration of a variety of marine animals, including piscivorous fishes^4, 6, 18–20^. Recent studies conducted in coastal waters using acoustic telemetry have demonstrated movement patterns of fish species over fine spatial scales to an accuracy of several m^21–24^. These acoustic telemetry systems can provide continuous detailed data on the position of tagged fish over months and years. The use of advanced technology, such as acoustic telemetry, in surveys of day/night movements of piscivorous fishes would be helpful for understanding diurnal changes in trophic flows in and around seagrass beds.

**Figure 2:**
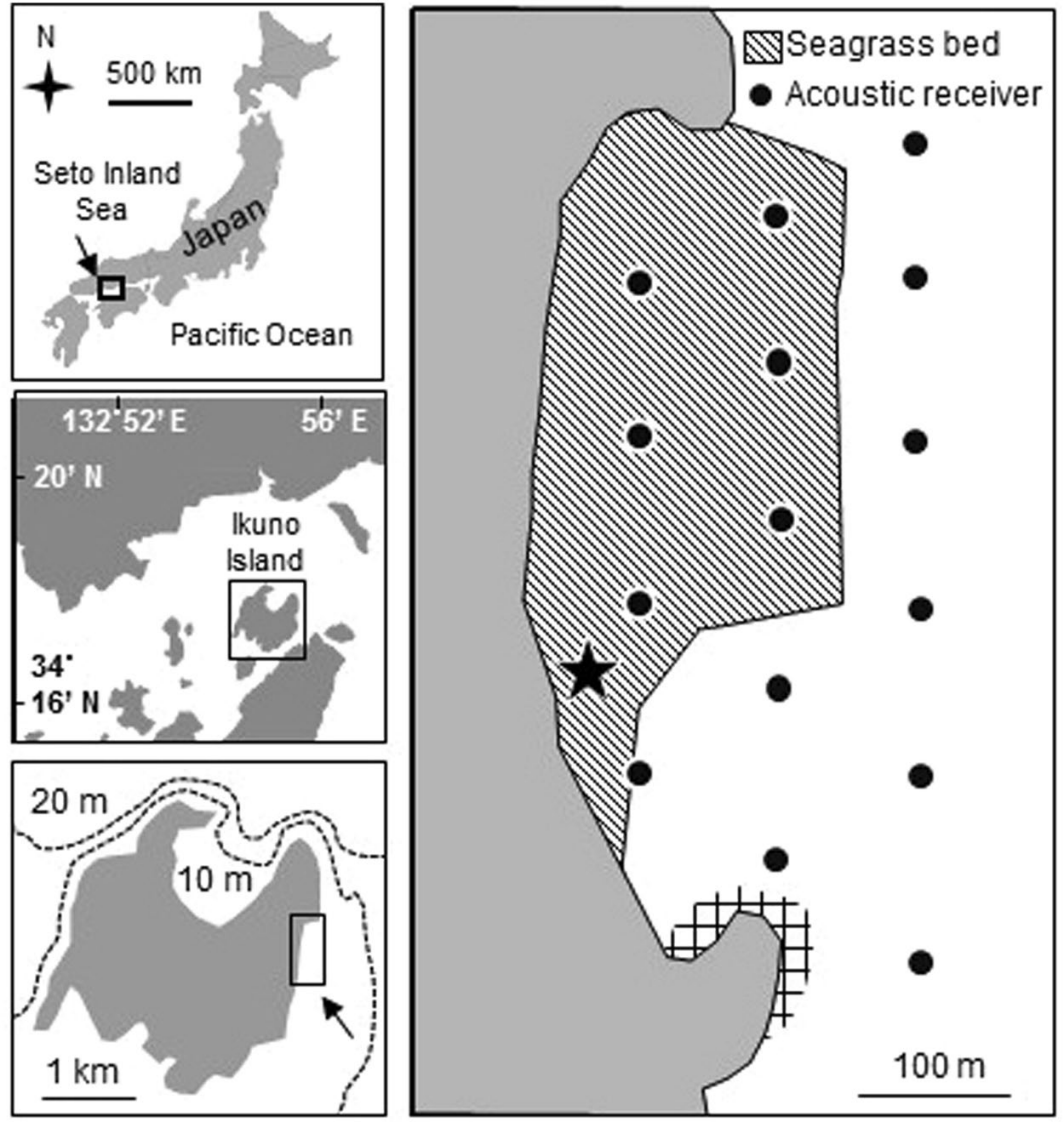
Map showing the survey area off Ikuno Island, the central Seto Inland Sea, southwestern Japan. Seasonal fish sampling was conducted in the seagrass bed during daytime and nighttime in October 2009, January, April, and July 2010. Depth contours are represented by dotted lines in the bottom-left panel. Acoustic telemetry was used to detect visits by predatory fishes in the same area. The star indicates the location where tagged fishes were released for acoustic telemetry. The rocky bottom area in the southern part of the surveyed area is indicated with a meshed shading. The maps were created by the first author using Microsoft PowerPoint 2010 (https://www.microsoft.com).

In the present study, diurnal and seasonal changes in fish community structures and trophic interactions were examined in a temperate seagrass bed (Fig. 2). Species richness, abundance and biomass of piscivorous fishes, and the trophic level of fish communities and predation rates of juvenile and small-sized fishes were compared between the day and nighttime over four seasons. Acoustic telemetry was applied to confirm whether nocturnal visits of piscivorous fish predators elevate the trophic level and predation rate at night in the seagrass bed. The ecological function of seagrass beds as a nocturnal feeding ground for piscivorous fishes was assessed to address the paradox from the perspective of the predators.

## Results

### Fish community in the seagrass bed

In total, 6,639 fishes belonging to more than 46 taxa in 25 families were collected from the seagrass bed during the seasonal samplings (Supplementary Table S1). *Acanthopagrus schlegelii* (27.9%), *Sebastes cheni* (19.0%), *Rudarius ercodes* (13.1%), *Plotosus lineatus* (7.4%), and *Favonigobius gymnauchen* (6.6%) were numerically dominant when the seasonal data were pooled.

**Figure 3:**
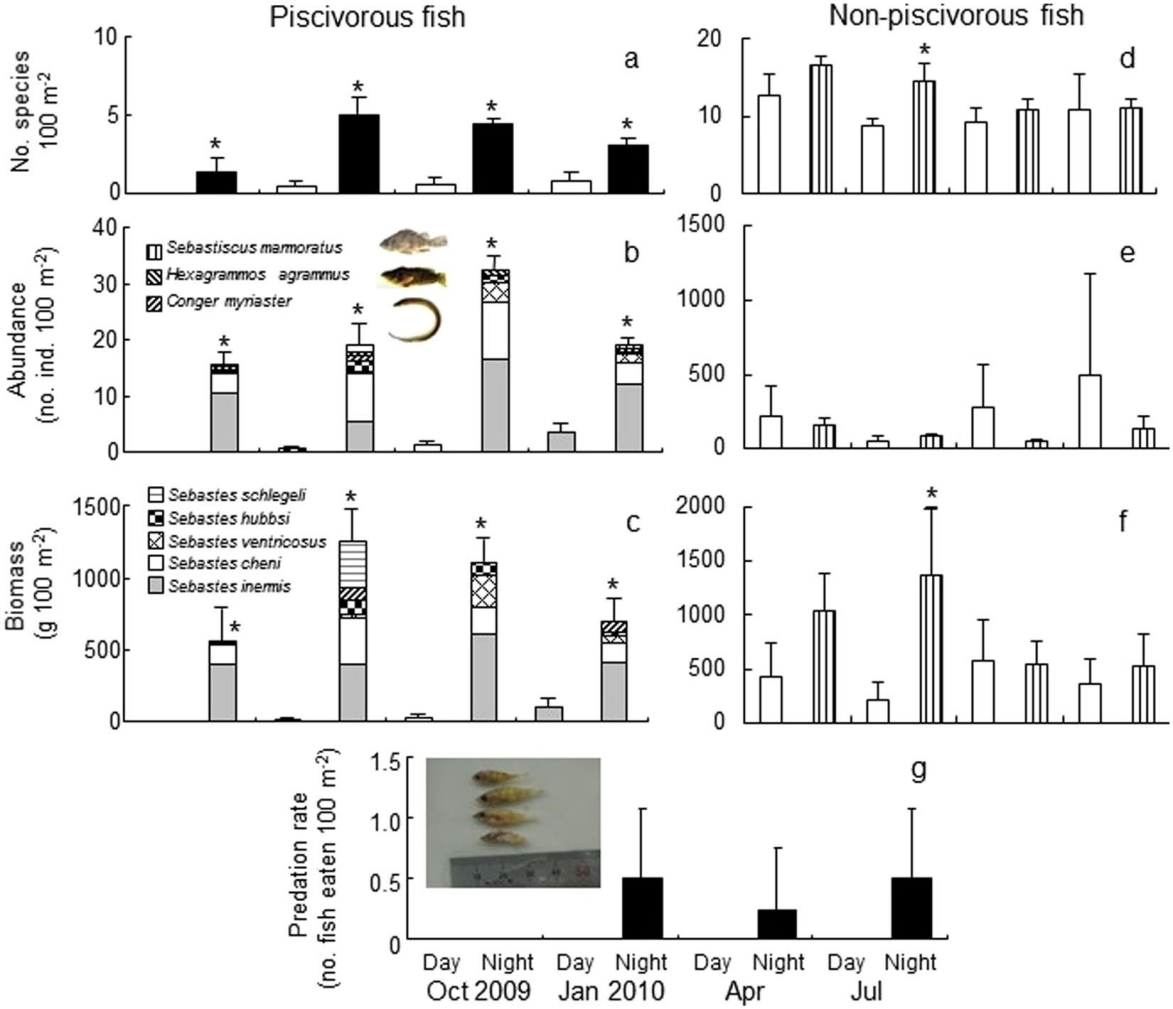
Comparison of the number of species (**a**), abundance (**b**), and biomass (**c**) of piscivorous fishes; the number of species (**d**), abundance (**e**), and biomass (**f**) of non-piscivorous fishes; and the occurrence of fish predation (**g**) between the daytime and nighttime during seasonal sampling from October 2009 to July 2010 in the seagrass bed. Vertical bars represent the standard deviation and the asterisk indicates a significant difference between the daytime and nighttime within the season (Wilcoxon test, *p* < 0.05). The photograph in the bottom panel shows rockfish (*Sebastes* spp.) juveniles found in the stomach of a predator (*Sebastes inermis*).

Seven piscivorous fish species were collected during the samplings (*Sebastiscus marmoratus, Sebastes schlegeli, Hexagrammos agrammus, Conger myriaster, Sebastes hubbsi, Sebastes ventricosus, Sebastes cheni* and *Sebastes inermis*). Mean (±standard deviation) number of piscivorous fish species (number 100 m^−2^) ranged between 0 (October 2009) and 1.0 ± 0.8 (July 2010) during the daytime, and between 2.0 ± 1.8 (October 2009) and 5.0 ± 1.5 (January 2010) during the nighttime (Fig. 3a: Wilcoxon test, *p* < 0.05 for all).

The mean abundance (number 100 m^−2^) of piscivorous fishes in the daytime was the highest in July 2010 (3.8 ± 5.0) and the lowest in October 2009 (0), and in the nighttime it was the highest in January 2010 (31.5 ± 6.2) and the lowest in October 2009 (15.3 ± 7.6), with significant differences observed between day and night in all seasons (Fig. 3b: Wilcoxon test, *p* < 0.05 for all). The mean biomass (g 100 m^−2^) of piscivorous fishes in the daytime was the highest in July 2010 (107.4 ± 148.1) and the lowest in October 2009 (0), and in the nighttime, was the highest in January (1261.1 ± 839.2) and the lowest in October 2009 (558.0 ± 360.4), with significant differences observed between the daytime and nighttime values in all seasons (Fig. 3c: Wilcoxon test, *p* < 0.05 for all).

The mean number of non-piscivorous fish species (number 100 m^−2^) ranged between 8.8 ± 1.0 (January 2010) and 12.8 ± 2.8 (October 2009) during the daytime, and between 10.8 ± 1.5 (April 2010) and 16.5 ± 1.3 (October 2009) during the nighttime (Fig. 3d: Wilcoxon test, *p* < 0.05 for January). The mean abundance (number 100 m^−2^) of non-piscivorous fishes in the daytime was the highest in July 2010 (500.0 ± 682.1) and the lowest in January 2010 (56.3 ± 26.9), and in the nighttime, was the highest in October 2009 (155.3 ± 56.0) and the lowest in April 2010 (50.0 ± 18.5), with no significant differences, observed between the daytime and nighttime values in all seasons (Fig. 3e: Wilcoxon test, *p* > 0.05 for all). The mean biomass (g 100 m^−2^) of non-piscivorous fishes in the daytime was the highest in April 2010 (570.5 ± 385.7) and the lowest in January 2010 (212.2 ± 161.7), and in the nighttime, was the highest in January (1368.0 ± 640.6) and the lowest in July 2010 (528.1 ± 295.1), with significant differences observed between day and night only in January (Fig. 3f: Wilcoxon test, *p* < 0.05).

### Day-night change in predation rate and trophic level

Examination of the stomach contents of potential predators (n = 182) revealed an increase in the predation rate during the night in three of the four seasons (Fig. 3g). No predation was observed during the day in any season whereas the nighttime predation rate (number of fish eaten by predators 100 m^−2^) ranged between 0 (October 2009) and 0.5 ± 0.6 100 m^−2^ (January and July 2010).

**Figure 4:**
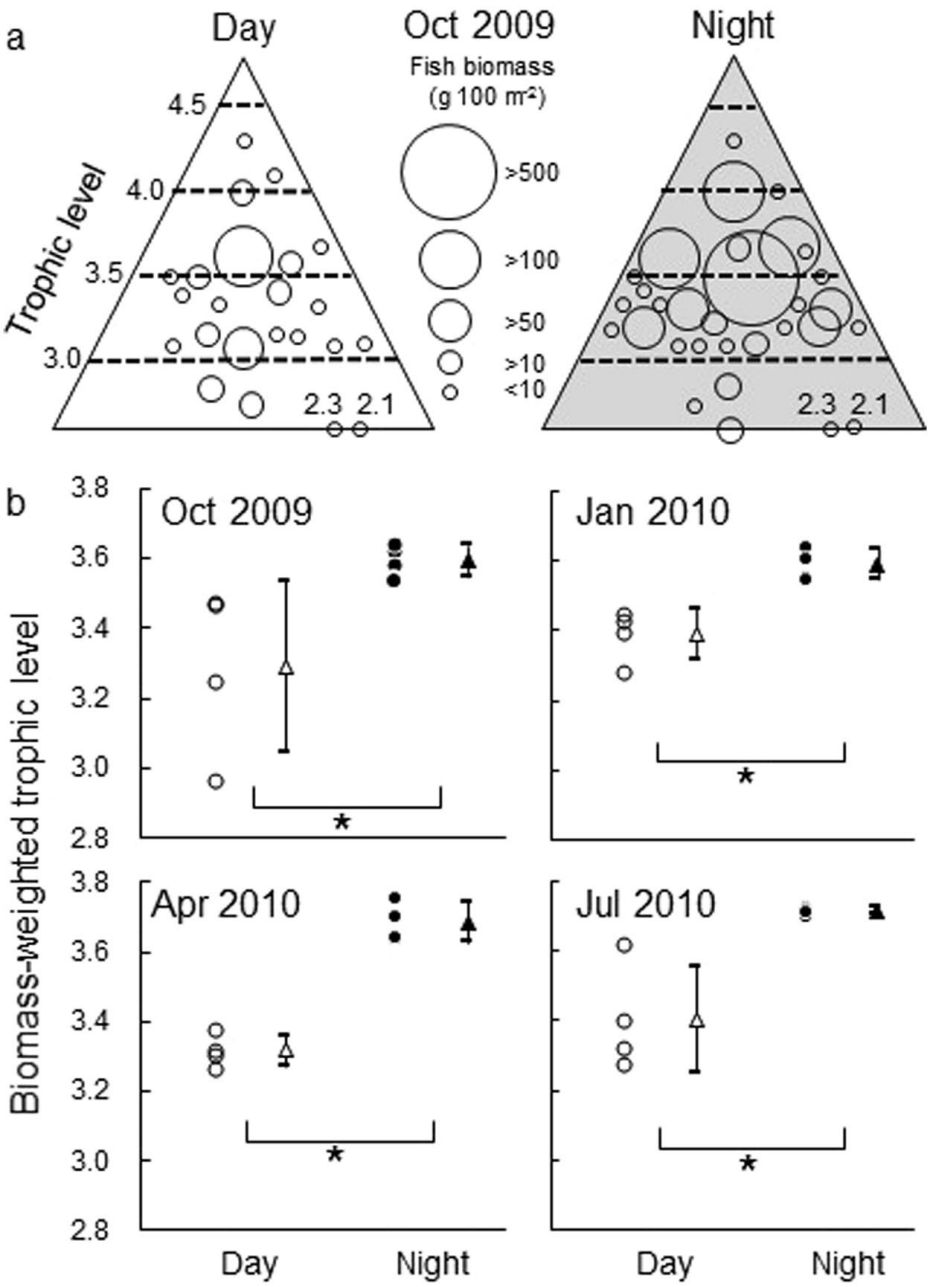
A schematic drawing of day/night changes in the trophic level and biomass (g 100 m^−2^) of each fish species in October 2009 (**a**), and a comparison of mean biomass-weighted trophic level between daytime (open symbols) and nighttime (closed symbols) in each season (**b**). Circles indicate the biomass-weighted trophic level calculated for each sampling trial, and triangles indicate the mean value (n = 4). The vertical bar represents the standard deviation and the asterisk indicates a significant difference between the daytime and nighttime within the same month (Wilcoxon test, *p* < 0.05). The trophic level of each fish species is summarized in Supplementary Table S1.

The biomass of the piscivorous fishes at trophic levels >3.0 increased during the nighttime (Fig. 4a). The biomass-weighted trophic level of the fish community during the night was significantly higher than that during the day in all seasons (Fig. 4b: Wilcoxon test, *p* < 0.05 for all).

### Nocturnal visits by piscivorous fishes

**Figure 5:**
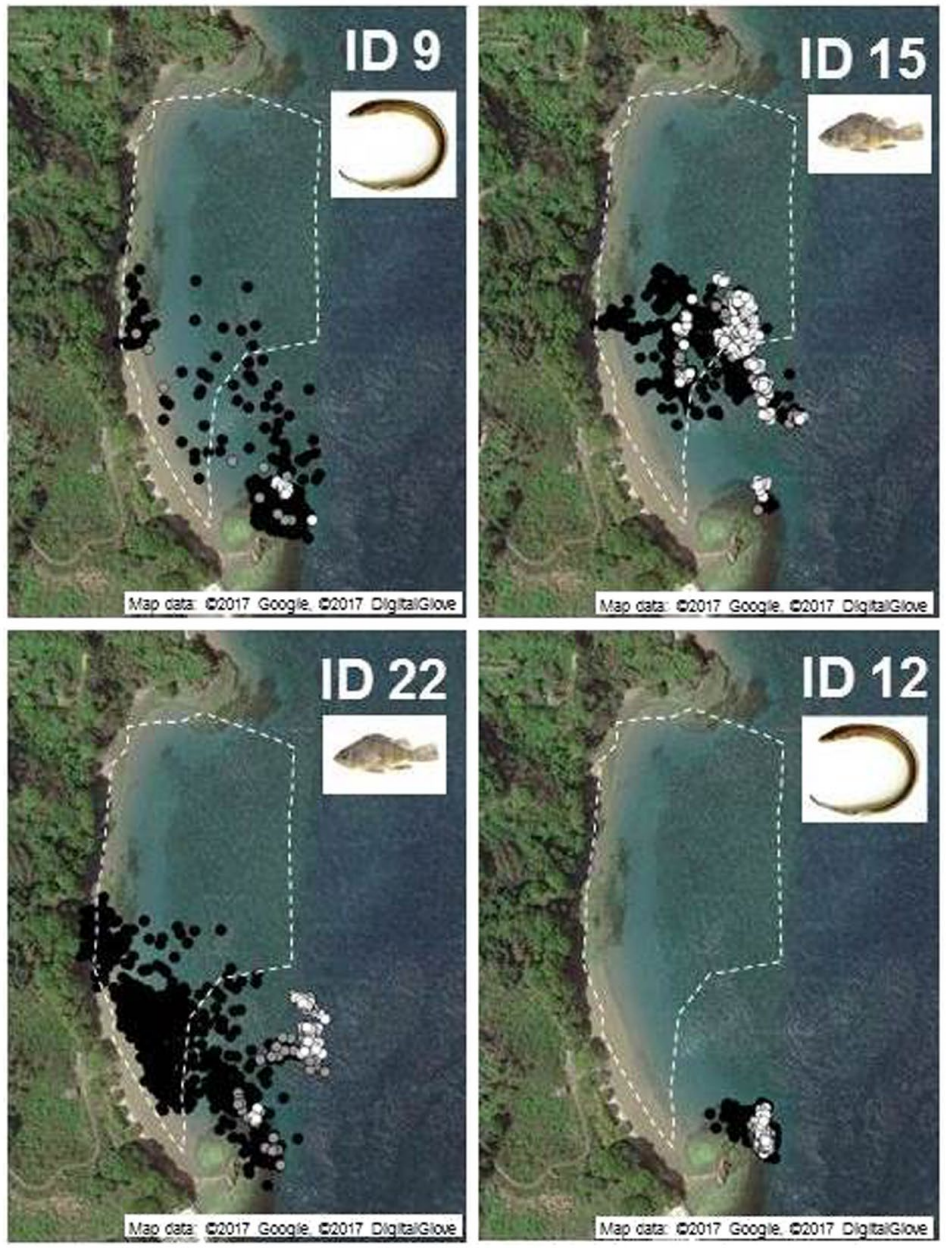
Typical movement patterns of piscivorous fishes revealed by acoustic telemetry in the seagrass bed. A conger eel (ID 9) and a rockfish (ID 22) during nocturnal visits to the seagrass bed (movement pattern type A); a rockfish that remained within the seagrass bed during both daytime and nighttime (ID 15: movement pattern type B); and a conger eel that remained at the rocky bottom area close to the seagrass bed during both the daytime and nighttime (ID 12: movement pattern type C) are shown. The seagrass bed is indicated by the dotted line. White, gray, and black circles indicate daytime (0500–1900 h), dawn/dusk (0500–0700/1700–1900 h), and nighttime (1900–0500 h). Information on the size and movement pattern of all fishes used for telemetry (n = 30) is summarized in Supplementary Table S2. Map data: ©2017 Google, ©2017 DigitalGlove.

Evidence of nocturnal visits by dominant piscivorous fishes in the seagrass bed was obtained by acoustic telemetry. Of 30 piscivorous fishes tagged, evidence of nocturnal visits to the seagrass bed (100% frequency of occurrence in the seagrass bed during the night, but being outside the seagrass bed during the day: migration type A; Supplementary Table S2) was obtained for 11 rockfishes (eight *S. inermis* and one *S. ventricosus*) and three conger eels, although some tended to stay within or outside the seagrass bed during the monitoring period (Fig. 5). Rockfishes had higher dependence on the seagrass bed, especially during 2015 when monitoring was performed for longer periods (types A and B: 86.4%), whereas the proportion of type C (no visit to seagrass bed) was 15.4%. The proportion of type C in conger eel was slightly higher than in rockfishes, in both 2014 (25%) and 2015 (20%).

### Environmental conditions of the seagrass bed

Water temperature ranged between 12.0 (January 2010) and 24.3 °C (October 2009) with minimal differences found between day and nighttime (a maximum difference of 1.8 °C within a day occurred in April 2009: Supplementary Table S1). Salinity ranged between 31.9 (July 2010) and 33.0 (January 2010) with a maximum difference of 1.8 within a day in July 2010. Mean seagrass shoot density was the highest in October 2009 (57.2 ± 12.4 m^−2^) and the lowest in January 2010 (32.0 ± 3.3 m^−2^: Supplementary Table S1). Mean seagrass canopy height was the highest in July 2010 (820.5 ± 133.0 mm) and the lowest in January 2010 (469.9 ± 111.6 mm). The effect of sampling season on seagrass shoot density and leaf length was significant (Kruskal-Wallis test, *p* < 0.05). Water temperature had a significant negative effect on the predator biomass (*p* = 0.048, multiple regression analysis) whereas the effect of seagrass shoot density was not significant (*p* = 0.069).

## Discussion

The increased frequency of occurrence and body size of large predators during the night in aquatic ecosystems have often been attributed to the higher catch efficiency of sampling gear^15–17^. In the present study, increase in piscivorous predator abundance and biomass in the seagrass bed at night were demonstrated in all seasons when collected using a fish net. In addition, the use of acoustic telemetry revealed nocturnal visits of piscivorous predators to the seagrass bed. We conclude that nocturnal visits by piscivorous predators increased the trophic level of the fish community in the seagrass bed during the night. In addition, juvenile and small-sized fishes were subjected to an elevated risk of predation during the night in the nursery area, which was previously thought to act as a predation refuge. The ecological function of seagrass beds as nocturnal feeding grounds for piscivorous fishes was confirmed by fish samplings coupled with acoustic telemetry surveys, and the paradox of the nursery habitat was highlighted from the the predator’s side.

Analysis of the movement of piscivorous fishes by acoustic telemetry indicated different visitation patterns to the seagrass bed within and among fish species (Table S2). The data obtained from fishes subjected to a longer monitoring period (>5 days) indicated that all rockfishes (n = 11) and 37.5% of conger eels (n = 3 of 8) made nocturnal visits to the seagrass bed. Rockfishes have been reported to visit and feed in seagrass, macroalgal beds, and rocky areas in shallow waters during the night^17, 25, 26^, which reflects a high dependence on vegetated or shallow habitats as nocturnal feeding grounds for these species. In conger eels, feeding on juvenile and small-sized fishes during the night has been demonstrated in seagrass and macroalgal beds^17, 26^, whereas their fishing grounds are widely distributed in shallow waters, including on sandy and muddy bottoms^27^. Therefore, the seagrass bed is suggested to act as a nocturnal feeding ground for conger eels.

Changes in the habitat complexity of the seagrass bed also affects its ecological function as a predation refuge for juvenile and small-sized fishes. The leaf shape and length of seagrass alters the fish community structure in tropical seagrass beds^28^. Natural tethering experiment and tank experiments revealed that the predation rates of juvenile fishes by piscivorous fishes were lower in seagrass beds^29, 30^. However, other research performed using ambush piscivorous fishes as predators showed that the habitat complexity of the seagrass bed did not always decrease predation rate^31^. A recent field survey provided evidence that the predation rate of juvenile rockfish in a macroalgal (*Sargassum* spp.) bed in the Seto Inland Sea was strongly affected by the abundance of piscivorous fishes visiting the nursery at night^26^. In the present study, the abundance of piscivorous fishes that visited the seagrass bed increased during the night in all seasons. In addition, more predators visited in winter and spring when seagrass shoot density was low. The seasonal fluctuation in nocturnal predator abundance was approximately twice in the seagrass bed. For the prey, the function of the seagrass bed as a predation refuge for juvenile and small-sized fishes was lower in winter and spring when the habitat complexity was low and predator abundance was high.

For the predators, analysis of diel and seasonal changes in non-piscivorous fishes indicated that the availability of the seagrass bed in the Seto Inland Sea did not significantly differ between seasons. Diel changes in the abundance and biomass of non-piscivorous fishes in the seagrass bed were less consistent compared to those of piscivorous fishes (Fig. 3). Significant differences in the number of species and amount of biomass of non-piscivorous fishes between day and night were observed only in January, whereas no significant differences in abundance were observed in any season. At night, there was a three-fold change in the magnitude of seasonal fluctuations in the mean abundance (56.3–500.0 100 m^−2^) and biomass (528.1–1368.0 g 100 m^−2^) of non-piscivorous fishes. Based on data for non-piscivorous fishes, seasonal variation in the ecological function of the seagrass bed as a nocturnal foraging ground for piscivorous fishes was small.

Factors that strongly affect predator-prey interactions in the seagrass bed include diel and developmental changes in physiological and behavioral aspects of both predator and prey. Juvenile rockfish (*S. cheni*), which are one of the most dominant non-piscivorous fishes in the seagrass bed in the Seto Inland Sea, feed^32^ and swim in schools^33^ only during the day. The rockfish juveniles form loosely aligned aggregations and maintain their body angle in inconsistent directions during the night under laboratory conditions^33^. Therefore, the ability of the juveniles to avoid predation through schooling behavior would be minimal, because the ability of the juveniles to school during the night is not well developed^33^. Therefore, rockfish juveniles are considered most vulnerable to predation by piscivorous fishes during the juvenile period. In general, school formation develops in juvenile fishes with increasing body length and the morphological development of sensory organs, such as eyes and lateral lines, indicating that vulnerability to predation decreases with their growth^34^. The size-selective predation (lower predation rate in larger fish) observed in natural habitats at night was explained by the development of antipredator behavior by rockfish juveniles through improvement in their schooling behavior^26^. Conversely, the piscivorous fishes that visited the seagrass bed at night in the present study (rockfishes and conger eel) have been reported to feed during the night^17, 25–27^. Differences in diel and developmental changes in morphological, physiological, and behavioral aspects of predator and prey are suggested to induce the ecological function of the seagrass bed as a nocturnal feeding ground for piscivorous fishes, which represents the predator’s side of the paradox.

The predation rate (0–2 fishes in the stomach of predators per sampling 100 m^−2^) estimated in the present study was comparable with that estimated with the same method for the most dominant fish species, juvenile *S. cheni*, at night in the surveyed area (1.3 100 m^−2^)^17^ and macroalgal (*Sargassum* spp.) bed off the Aba Island, 5 km northeast of the Ikuno Island (0–5.0 100 m^−2^)^26^. Another recent study on the mortality of seven cohorts of the dominant juvenile *S. cheni* in the macroalgal bed off Aba Island showed mortality coefficients between 0.031–0.048 for approximately two months during the post-settlement period^35^. The mortality coefficients of rockfish juveniles correspond to a loss of 3.0–4.7 of 100 juvenile rockfish d^−1^ 100 m^−2^. Assuming that juvenile rockfish predation mostly occurs during the night, and that fish prey eaten by piscivorous fish are evacuated from predator’s stomach multiple times each night, these estimates for juvenile mortality rate seem approximate.

In future studies, examination of the vital rates of predators, such as temperature-dependent behavior and gastric evacuation rate, will help to determine a more absolute consumption rate and its spatiotemporal variability. Information on the feeding behavior of piscivorous fishes at finer spatial and temporal scales, which could be monitored by acceleration data-logger/transmitter, should provide strong evidence for food consumption of predators within seagrass beds. Furthermore, seasonal and latitudinal variability in day/night lengths would affect the vital rates of each species and species interactions^32^. Annual variability in day/night lengths is greater at high latitudes, where daytime dominates in summer and nighttime dominates in winter. Information on latitudinal variability in fish community structures and predator-prey interactions would also permit a more comprehensive understanding of the spatio-temporal variability in the food web.

## Methods

### Field sampling

Biological and physical surveys were conducted on a seagrass bed off the eastern Ikuno Island, central Seto Inland Sea, Japan (Fig. 2). Ikuno Island has a population of approximately 30, with on human habitation on the eastern coast; therefore, the effects of human activities, such as industrialization and fishing, on the seagrass bed are minimal. The vegetation of the seagrass bed is dominated by the seagrass *Z. marina*, and the mean shoot density of this plant around the sampling site fluctuates between 20 and 160 m^−2^ throughout the year^36^. The bottom of the seagrass area is comprised of mud and sand. The assemblage of small (<100 mm TL) fish is dominated by filefish *Rudarius ercodes* in autumn (October 2008), sand goby *Favonigobius gymnauchen* in winter (January 2009), black rockfish *S. inermis* in spring (April 2009), and black sea bream *Acanthopagrus schelegeli* in summer (July 2009)^36^.

Seasonal fish sampling (October 2009, January, April, and July 2010) was conducted using a round seine net (2 m high, 30 m long, and 4 mm mesh aperture) using previously described method^35, 36^ in the day (1100–1700 h: October 6, 2009, January 7, 2010, April 15, 2010, and July 9, 2010) and nighttime (1930–0300 h: October 7, 2009, January 6, 2010, April 14, 2010, and July 4, 2010). Fish were collected from four separate locations, randomly selected within the seagrass bed during day and night samplings. Each fish collection covered an area of 100 m^2^. Tidal levels ranged from 50–130 cm during the sampling. Collected fish were preserved in 10% formalin seawater solution. The temperature and salinity of surface water were measured at each sampling. Seagrass shoot density was measured in at least four randomly placed 0.5 m square quadrats in the seagrass bed. The length of seagrass leaves from at least ten shoots were measured in the daytime sampling, during each sampling season.

### Laboratory procedures

Fish were identified to the lowest possible taxon^37, 38^ and were measured to determine their total length (mm) and wet weight (g). Mean fish abundance and biomass were expressed as the number and wet weight of fish per 100 m^2^ for piscivorous and non-piscivorous fish groups, based on the stomach content analysis in the present study and Fishbase (www.fishbase.org/). The stomach contents of piscivorous fishes (n = 182, as potential predators^37^) were identified, counted, and weighed. Juveniles of these piscivorous fish species (<1 year old) were not considered as potential predators because they are not piscivorous. Predation rate was expressed as the number of fishes eaten per 100 m^2^. To compare the trophic level of the fish community between day and night, the biomass-weighted trophic level was calculated for each sampling trial. The biomass-weighted trophic level was compared between the day and night by sampling season using the Wilcoxon test. To detect possible effects of the environmental conditions on predators, a multiple regression analysis was conducted with water temperature and seagrass shoot density as explanatory variables and predator biomass as a dependent variable.

### Acoustic telemetry

Based on the list of dominant piscivorous fishes in the surveyed area^17, 36^, 21 rockfishes (*S. inermis*: n = 20, *S. ventricosus*: n = 1) and nine conger eels (*C. myriaster*: n = 9) were used in the telemetry survey (Supplementary Table S2). The rockfishes were captured on the seagrass bed using round seine net by the same method used to sample fish for the community survey. The conger eels were captured by basket traps. A recovery period of 1–3 days was allowed prior to tagging. The fish were tagged with acoustic transmitters (69 kHz, V9-1H 151 dB, battery life: 264 days, average signal interval: 110–250 s; V9A-2H, 151 dB, battery life: 154 days, average signal interval: 110–250 s, Vemco Inc. Halifax). The transmitters were surgically implanted into the peritoneal cavity of the fish under anesthesia with 0.1% 2-phenoxyethanol^39^. The fish were then returned to a holding tank containing aerated seawater sampled from the vicinity of their capture site and allowed to recover. No mortalities occurred during tagging. Preliminary experiments using dummy transmitters demonstrated that the process of intraperitoneal implantation had no discernible effects on the feeding or swimming behavior of rockfish and conger eel over a period of approximately 1 month. The tagged fish were released at the shallow area of the coast (ca. 1 m depth, edge of the seagrass bed where the fish were collected: Fig. 2) after tagging.

The tagged fish were monitored after their release on July 26, 2014 and May 22, 2015 by the Vemco positioning system using 15 fixed monitoring receivers (VR2W, Vemco Inc.)^40, 41^ and 15 stationary synchronization tags (V16-5H, signal interval: 600 s, Vemco Inc.). Receivers (detection range: 200–400 m) with synchronization tags were deployed 80–100 m apart (hereafter VPS array: Fig. 2). The VPS array covered the seagrass bed and neighboring rocky area to the south. This system accurately provides the fine-scale horizontal position (error: several m) using the technique of time-difference-of arrival (TDOA), or hyperbolic positioning^40, 41^. The TDOA of a signal to a given pair of receivers generates a set of potential source locations as a hyperbola. Then, the exact source coordinates on the horizontal plain can be calculated as an intersection point of the multiple hyperbolas derived from the TDOAs. The TDOA is the most effective parameter for the accurate localization. The tagged fish were monitored after their release by the VPS until October 27, 2014 and October 20, 2015.

The time spent by each tagged fish within the seagrass bed during the nighttime (1900–0500 h) was summed. Data with horizontal position error (HPE) <10 were processed to analyze the predators’ movement. The HPE is a relative measurement, and a calculated position with higher HPE provides less accurate information than one with a lower HPE^30, 31^. As a conservative precaution, data obtained for the first two days after release were not used in this analysis because the tagged fish, especially *Sebastes* species, might remain in the seagrass area immediately after release^39^. If the time lapse between consecutive data recorded in each period was less than 60 min, the fish was recorded to have stayed continuously in the seagrass bed. Time spent in the seagrass bed was not summed if the tagged fish continuously stayed in the seagrass area for more than one day. The movement patterns of the tagged fishes were grouped into three types (Supplementary Table S2); A: nocturnal visit to the seagrass bed with 100% frequency of occurrence at night in the seagrass bed but outside the seagrass bed during the day, B: remain in the seagrass bed, C: no visit to the seagrass bed and D: tag shed or died/preyed upon just after release.

### Ethical statement

All applicable institutional and/or national guidelines for the care and use of animals were followed. The procedures and protocols followed the guidelines of the Committee for Animal Experiment of Hiroshima University (CD001651) and those for the use of fishes in research by the Ichthyological Society of Japan (http://www.fish-isj.jp/english/guidelines.html). No ethic or law violations are included in the present study.

## Electronic supplementary material

Supplementary Information
