# Supplementary material for: Increase in predation risk and trophic level induced by nocturnal visits of piscivorous fishes in a temperate seagrass bed
Source: Sci Rep. 2017 Jun 20;7:3895. doi: 10.1038/s41598-017-04217-3 (PMC5478620; doi:10.1038/s41598-017-04217-3)
Supplement: Supplementary file 1 — Supplementary Information [file 41598_2017_4217_MOESM1_ESM.pdf]

Supplementary information

Jun Shoji, Hiromichi Mitamura, Kotaro Ichikawa, Hikari Kinoshita, Nobuaki Arai.

Increases in predation risk and trophic level induced by nocturnal visit of piscivorous fishes in a temperate seagrass bed

Supplementary information 1.

Summary of environmental conditions and fish collected in the seagrass bed off Ikuno Island, central Seto Inland Sea, Japan, during the seasonal sampling from October 2009 to July 2010. Seagrass vegetation (shoot density and canopy height), water temperature and salinity are shown as environmental conditions. Total number of fish collected during day and night samplings, rank of 10 dominant taxa and trophic level (TL) are indicated for each species. The order of family name in the table is based on Nakabo (2002).

	Sampling period	Oct 2009		Jan 2010		Apr 2010		Jul 2010				
Environmental conditions	Seagrass shoot density (N m^{-2})	57.2±12.4		32.0±3.3		45.2±12.4		53.2±8.8				
	Seagrass canopy height (mm)	619.8±50.8		469.9±111.6		679.6±128.3		820.5±133.0				
		Day	Night	Day	Night	Day	Night	Day	Night			
	Water temperature	24.3	22.3	12.0	12.3	13.1	11.3	22.1	20.4			
	Salinity	32.3	32.4	33.0	33.1	32.2	32.0	31.9	30.1			
Fish family	Species									Total	Rank	TL
Congridae	*Conger myriaster*		3		3		1		3	10		4.0
Plotosidae	*Plotosus lineatus*	293	168		12				16	489	4	3.6
Plecoglossidae	*Plecoglossus altivelis altivelis*					3				3		2.8
Atherinidae	*Hypoatherina valenciennei*		1						181	182	7	3.2
Hemiramphidae	*Hyporhamphus sajori*		1		17				1	19		3.4
Aulorhynchidae	*Aulichthys japonicus*	17	4	10	15					46		3.1
Syngnathidae	*Syngnathus schlegeli*	8	6	6	4	4	9			37		3.1
	Hippocampus coronatus	2	3			1				6		3.3
Scorpaenidae	*Sebastiscus marmoratus*								2	2		3.6
	Sebastes inermis < 1 yr.	4	9		28	38	6	4	17	106		3.2
	S. inermis ≧ 1 yr.		42		22		66	14	49	193	6	4.0
	S. ventricosus < 1 yr.					160	5	5	2	172	9	3.2
	S. ventricosus ≧ 1 yr.		1		1	1	15		7	25		3.7
	S. cheni < 1 yr.	21	43	1	35	819	32	103	96	1,150	2	3.2
	S. cheni ≧ 1 yr.		14		34	5	40	1	14	108		3.7
	S. Schlegelii				5			1		6		3.8
	S. oblongus	1	1							2		3.7
	S. hubbsi		1	2	8		4		1	16		3.5
Synanceiidae	*Inimicus japonicus*						1			1		4.2
Tetrarogidae	*Hypodytes rubripinnis*	4	26	4	16	13	11	1		75		3.2
Hexagrammidae	*Hexagrammos agrammus*		1	3	3		3	1		11		3.3
	H. otakii					20	9		1	30		3.8
Cotiidae	*Pseudoblennius cottoides*		1	4	1	11	12	4		33		4.3
	P. percoides							2		2		4.1
Sillaginidae	*Sillago japonica*	2	44	3	1	1	1	3	3	58		3.5
Sparidae	*Acanthopagrus schlegelii*							1,739	112	1,851	1	3.2
	Pagrus major								2	2		3.7
Embiotocidae	*Ditrema temmincki temmincki*	2		16	54	2	13	48	45	180	8	3.5
Mugillidae	*Mugil cephalus*		2					18	0	20		2.5
Labridae	*Halichoeres poecilopterus*	10						1		11		3.6
Zoarcidae	*Zoarchias glaber*					1				1		3.2
Blenniidae	*Petroscirtes breviceps*	36	11		3					50		2.1
Callionymidae	*Repomucenus beniteguri*	2		2	1		1	1		7		3.3
Gobiidae	*Chaenogobius gulosus*			1				1		2		3.4
	Gymnogobius heptacanthus				1		1			2		3.2
	Sagamia geneionema		1	1	1					3		3.2
	Pterogobius elapoides					1	8	4		13		3.0
	Acanthogobius flavimanus	1				46	43	50	23	163	10	3.4
	Favonigobius gymnauchen	277	17	31	33	10	40	5	26	439	5	2.7
	Acentrogobius spp.	4	1	1	9			5	4	24		3.1
	Tridentiger trigonocephalus			3		1	1		1	6		3.3
Siganidae	*Siganus fuscescens*	12	13							25		2.3
Pleuronectidae	*Pleuronectes yokohamae*		1		1					2		3.3
Monacanthidae	*Rudarius ercodes*	396	170	139	68	51	34	12	2	872	3	3.1
	Thamnaconus modestus	1	75							76		3.4
	Stephanolepis cirrhifer	2	2							4		2.8
Tetraodontidae	*Takifugu pardalis*		4	1	1	14	13		1	34		3.3
	T. poecilonotus		1		1					2		3.2
	T. niphobles		15	3	22	4	22		2	68		3.3
	Total	1,095	682	231	400	1,206	391	2,023	611	6,639		

Supplementary information 2.

Information on the piscivorous fishes used for acoustic telemetry. Total length, body weight, date of release, monitoring period and movement pattern were indicated. Frequency of nighttime (1900-0500 h) stay in the seagrass bed (%) as an index of nocturnal visit of the piscivorous fishes to the seagrass bed was calculated by dividing the number of days remaining in the seagrass bed during nighttime (N) by the number of days monitored in the seagrass bed (S). Movement pattern was categorized to A: nocturnal visit to the seagrass bed with 100% of frequency of occurrence during nighttime in the seagrass bed but outside the seagrass bed during daytime; B: remain in the seagrass bed; C: no visit to the seagrass bed; D: tag shed or died (predated) just after release.

Fish ID	Species	Total length (mm)	Body weight (g)	Date of release	Monitoring period (d)	N of days monitored in seagrass bed (S)	N of days monitored during daytime in seagrass bed	N of days monitored during nighttime in seagrass bed (N)	Frequency of the nighttime monitirong in seagrass bed (N/S*100, %)	Averege (±SD) duration of the nighttime monitoring in seagrass bed (min/d)	Movement pattern
1	*S. inermis*	175	100	26-Jul-2014	1	0	0	0	0		C
2	*S. inermis*	163	79	26-Jul-2014	2	0	0	0	0		C
3	*S. inermis*	159	73	26-Jul-2014	2	0	0	0	0		C
4	*S. inermis*	164	68	26-Jul-2014	3	0	0	0	0		C
5	*S. inermis*	158	63	26-Jul-2014	2	0	0	0	0		C
6	*S. inermis*	145	52	26-Jul-2014	2	0	0	0	0		C
7	*S. inermis*	136	46	26-Jul-2014	2	0	0	0	0		C
8	*S. inermis*	214	146	26-Jul-2014	2	0	0	0	0		C
9	*C. myriaster*	626	403	26-Jul-2014	89	4	0	4	100	199±99	A
10	*C. myriaster*	553	310	26-Jul-2014	94	0	0	0	0		C
11	*C. myriaster*	465	130	26-Jul-2014	1	0	0	0	0		C
12	*C. myriaster*	555	269	26-Jul-2014	94	0	0	0	0		C
13	*S. inermis*	228	201	22-May-2015	98	71	0	71	100	358±154	A
14	*S. ventricosus*	242	212	22-May-2015	64	56	0	56	100	305±133	A
15	*S. inermis*	181	96	22-May-2015	27	25	25	25	100	whole period	B
16	*S. inermis*	196	126	22-May-2015	6	4	4	4	100	whole period	B
17	*S. inermis*	203	137	22-May-2015	17	7	0	7	100	296±84	A
18	*S. inermis*	193	117	22-May-2015	10	8	0	8	100	300±157	A
19	*S. inermis*	185	109	22-May-2015	121	67	0	67	100	246±131	A
20	*S. inermis*	175	78	22-May-2015	8	6	0	6	100	294±102	A
21	*S. inermis*	166	68	22-May-2015	64	43	0	43	100	197±102	A
22	*S. inermis*	151	58	22-May-2015	97	77	0	77	100	318±125	A
23	*S. inermis*	152	62	22-May-2015	1	0	0	0	0		D
24	*S. inermis*	145	50	22-May-2015	122	116	98	116	100	624±151	A/B
25	*S. inermis*	180	91	22-May-2015	1	0	0	0	0		D
26	*C. myriaster*	596	340	22-May-2015	71	0	0	0	0		C
27	*C. myriaster*	501	288	22-May-2015	73	0	0	0	0		C
28	*C. myriaster*	607	359	22-May-2015	83	0	0	0	0		C
29	*C. myriaster*	628	372	22-May-2015	84	66	8	66	100	176±124	A/B
30	*C. myriaster*	459	155	22-May-2015	122	120	120	120	100	whole period	B
